# Design of a Smart Conducting Nanocomposite with an Extended Strain Sensing Range by Conjugating Hybrid Structures

**DOI:** 10.3390/polym14132551

**Published:** 2022-06-23

**Authors:** Byung-Ho Kang, In-Yong Jeong, Sung-Hoon Park

**Affiliations:** Department of Mechanical Engineering, Soongsil University, 369 Sangdo-ro, Dongjak-Gu, Seoul 06978, Korea; royce2080@naver.com (B.-H.K.); jiy5802@daum.net (I.-Y.J.)

**Keywords:** polymer composite, carbon nanotube, strain sensor, hysteresis, plastic deformation

## Abstract

In recent years, flexible and wearable strain sensors, consisting of a polymer matrix and a conducting filler, have received extensive attention owing to their physical advantages, such as being lightweight, stretchable, and having the potential for application to complex forms. However, achieving a low hysteresis of the relative change in resistance, wide sensing range, and reduced plastic deformation is still challenging. To address these issues, in this study, we developed hybrid conducting composites with a wide range of sensing abilities and low hysteresis. The bi-layer composites, comprising a carbon nanotube (CNT) composite layer with reinforced/conducting properties, and a natural rubber-based layer with extreme strain properties, could effectively circumvent their limitations. Compared to single-layer CNT composites, the bi-layer structure could increase the tensile strain with reduced plastic deformation, resulting in the prevention of surface cracks on the CNT composite. In addition, it has the benefit of measuring a wider sensing range, which cannot be measured in a single-CNT composite system. A cyclic stretching/releasing test was performed to demonstrate that the strain sensor exhibited excellent reproducibility. Our results can function as a useful design guide for stretchable sensor applications.

## 1. Introduction

Research on flexible strain sensors has been extended to commercialize wearable devices capable of tracking human motions [1,2,3,4,5]. Because conventional strain sensors are made of metal, various materials for stretchable strain sensors are being introduced. In addition, many studies are focusing on improving stretchability, durability, and repeatability, and lowering hysteresis. Since transformation into various forms is essential if a sensor is to recognize human movements, there have been comprehensive studies on flexible and stretchable polymer-based composites for sensor applications [6,7,8,9,10]. Furthermore, because elastomeric polymers are electrically insulating, electrically conducting fillers must be introduced for piezoresistive system-based sensors.

Commonly used conductive fillers include carbon nanomaterials, such as graphene [11,12], carbon black (CB) [13,14,15], carbon nanotubes (CNTs) [16,17,18,19,20,21], and Ag nanowires (Ag NW) [22,23]. Among them, CNTs are 1D materials with a high aspect ratio; therefore, even if the range of strain increases, the electrical network can be maintained, which is advantageous for a stretchable piezoresistive system [2]. CNTs can be classified into single-walled CNTs (SWNTs) and multi-walled CNTs (MWNTs), based on the number of walls. Because CNTs have excellent mechanical strength, electrical conductivity, and thermal conductivity, they have the potential to be utilized in various applications [24,25,26,27,28].

Recently, new attempts have been initiated to meet the requirements of stretchable devices, such as a wide sensing range, stability, and low hysteresis, by modifying the composite structure. In particular, there has been an increase in studies on the fabrication of hybrid-structured composites covering the elastomeric polymer layer above and below the conducting layer [2,3,4,5,29]. However, in practical terms, the analysis of the effect of such a hybrid-structured system compared with the existing single composite is insufficient.

Therefore, we analyzed the advantages of a hybrid-structured composite compared to a single composite from the perspective of hysteresis and strain range. The bi-layered composite was prepared by sealing natural rubber (NR) with excellent tensile strain (<500%) onto MWNT/NR with excellent electrical conductivity in a single composite. NR was selected as the base polymer matrix and hybrid-structure layer owing to its ultra-stretchability and biocompatibility. The effect of the pure NR layer was analyzed with MWNT contents of 1 and 5 wt.%. In addition, the adhesion between homogeneous polymers was excellent, according to the cross-sectional image after tensile strain. This bilayer composite could lower the hysteresis of the change in the initial resistance, compared to the single composite system, because the NR layers prevent surface cracks from forming on the conducting layer due to strain. In addition, this hybrid structure can reduce plastic deformation and widen the sensing range. Furthermore, this system showed excellent repeatability and durability in a cyclic test (130% strain, 100 cycles) in the 5 wt.% sample.

## 2. Materials and Methods

### 2.1. Materials

Natural rubber (NR) purchased from InfoChems (RSS#3, Goyang City, Gyeonggi-do, Korea) was adopted as an elastomeric matrix. Multi-walled carbon nanotubes (MWNT, JEIO, Incheon, Korea) were used as conductive fillers. This MWNT had a diameter of 5–7 nm, a bundle length of 50–150 μm, and purity of >96.5 wt.%. Dicumyl peroxide (DCP), used as a crosslinking agent, was purchased from Sigma Aldrich (St. Louis, MO, USA), and chloroform, used as a solvent for NR and MWNT, was purchased from DAEJUNG Chemicals (Siheung, Korea).

### 2.2. Fabrication of MWNT/NR Single Composite and MWNT/NR Bi-Layer Composite

Both the MWNT/NR single composite and the MWNT/NR bi-layer composites with low contents (1 wt.%) and high contents (5 wt.%) were fabricated using the ultrasonication method. Because the dispersion of MWNT is essential in manufacturing the composite, a solution process using an ultrasonicator (Sonics & Materials Inc., Newtown, CT, USA, VC 505) was performed.

First, 5 g of NR was dissolved in 250 mL of chloroform using a magnetic stirrer (IKA, C- MAG HS7) at 45 °C, 500 rpm for 24 h. The MWNTs were then mixed with 150 mL of chloroform in another beaker for uniform dispersion, and the first ultrasonic dispersion was performed at 200 W for 30 min. Next, a secondary ultrasonic dispersion was performed for 1 h by adding an NR solution to the CNT dispersion, which was then sufficiently mixed. Then, 2 wt. % DCP was added to the resultant dispersion and mixed for 30 min. Finally, the solvent was evaporated in a vacuum oven at 45 °C for 16 h. Because NR is a thermosetting polymer, the obtained MWNT/NR film was pre-pressed at 3 MPa for 2 min at 170 °C in a 0.5 mm mold, and then pressed at 15 MPa for 18 min. This process fabricated 0.5 mm of the MWNT/NR single composite.

**Figure 1:**
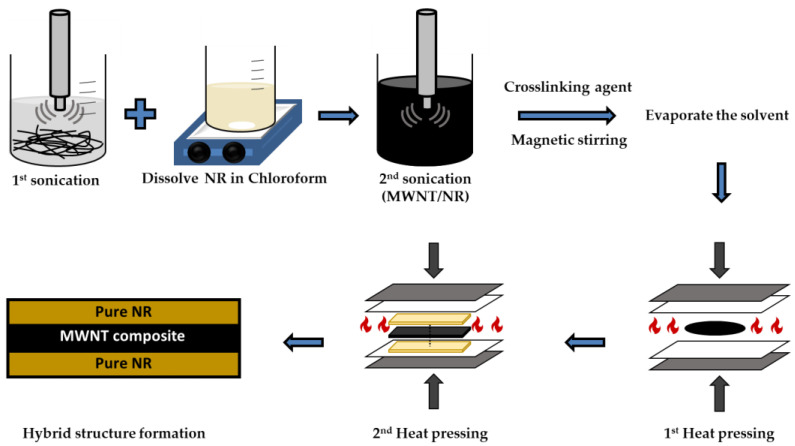
Scheme of the fabrication method of the MWNT/NR single composite and the MWNT/NR hybrid structure.

The NR film, previously prepared to fabricate the MWNT/NR bilayer composite, was placed at the upper and lower ends of the single composite and heat-pressed in the same manner as mentioned above. In the case of the bilayer composite, a 1.5 mm mold was used to design all layers with thicknesses of 0.5 mm. This process is illustrated in Figure 1.

### 2.3. Characterization

To confirm the MWNT/NR dispersion and interface adhesion of the bilayer composite, the composite cross-section was examined by scanning electron microscopy (SEM, ZEISS Inc., New York, NY, USA, Gemini SEM 300). SEM was operated under an accelerated voltage of 5 kV, and a cryo-fractured sample was prepared to obtain a cross-sectional view of the composite. In addition, to measure the interfacial adhesive force, the broken cross-section, which was coated with Pt, was measured after tensile strain.

A 2-wire mode multimeter (DMM7510, Keithley, Solon, OH, USA) was used to measure the electrical conductivity of the MWNT/NR composite. First, an electrode was formed with Ag paste (Protavic, Levallois-Perret, France) after 5 min of UV etching to reduce contact resistance. Then, the Ag electrode was crosslinked in a convection oven at 130 °C for 1.5 h.

The mechanical properties, such as Young’s modulus and elongation, were measured using a universal testing machine (UTM, DRTECH, Seongnam-si, Korea). The specimen used for the UTM test was a linear-type sample with a dimension of 5.0 × 50.0 × (0.5 for single composite, 1.5 for bi-layer composite) mm^3^. All of the samples were measured at the same speed of 20 mm/min.

Finally, the dynamic strain test of the composite, according to the uniaxial strain, was performed using a 3D stretching machine (3D-SM, NAMIL Optical Instruments Co., Hongkong, China). For the bilayer composite, Cu tape was attached inside the Ag paste to form a long electrode. Hysteresis analysis was performed in the range of 50–100% in all samples, and a continuous stretching/releasing test was performed for 100 cycles for repeatability and durability analysis.

## 3. Results and Discussion

### 3.1. Morphology Analysis

**Figure 2:**
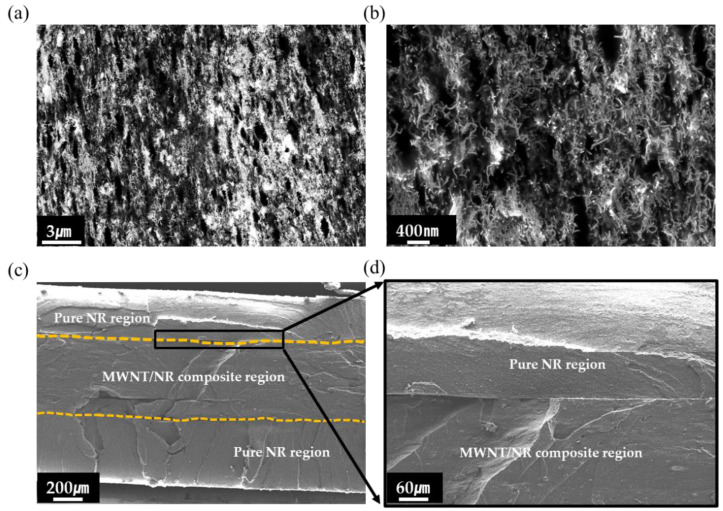
SEM image of cryo-fracture surface of 5 wt.% MWNT/NR composite: (**a**) low magnification, and (**b**) high magnification. Cross-section view of hybrid structure: (**c**) low magnification, and (**d**) middle magnification.

Figure 2 shows SEM images of the cryo-fractured surfaces of the 5 wt.% MWNT/NR composite, and the excellent adhesion interfaces of the bilayer composite. Because CNTs exist as bundles by van der Waals forces, a well-dispersed state must be obtained when used as a conductive filler in composites. Because obtaining well-dispersed high contents of MWNTs in the composite is more challenging than it is with low contents, measuring the dispersion state of high contents is essential. Therefore, we measured the dispersion state image of the 5 wt.% MWNT/NR composite at low and high magnifications, as shown in Figure 2a,b, respectively. As shown in Figure 2a,b, we obtained uniformly dispersed MWNTs in the composite by ultrasonication. Ultrasonication is a useful method for dispersing nanofillers in a matrix in a solution process [30,31]. Therefore, our sonication operating conditions (200 W, 2-step process) were excellent for dispersing MWNTs in the NR matrix.

We then measured the adhesion between the NR layer and the 5 wt.% MWNT/NR composite using SEM images. Because the absence of cracks on the interface after stretching indicates that the NR and MWNT/NR layers are compatible, a tensile broken sample was prepared. As shown in Figure 2c, there were no cracks at the interfaces, indicating that adhesion between the homogenous polymer systems was successful. For precise analysis, a high-magnification image of the interface was measured, as shown in Figure 2d.

Furthermore, the thicknesses of the MWNT/NR composite and pure NR regions were calculated to be ~500 μm.

### 3.2. Electrical Conductivity and Electrical Percolation Theory

**Figure 3:**
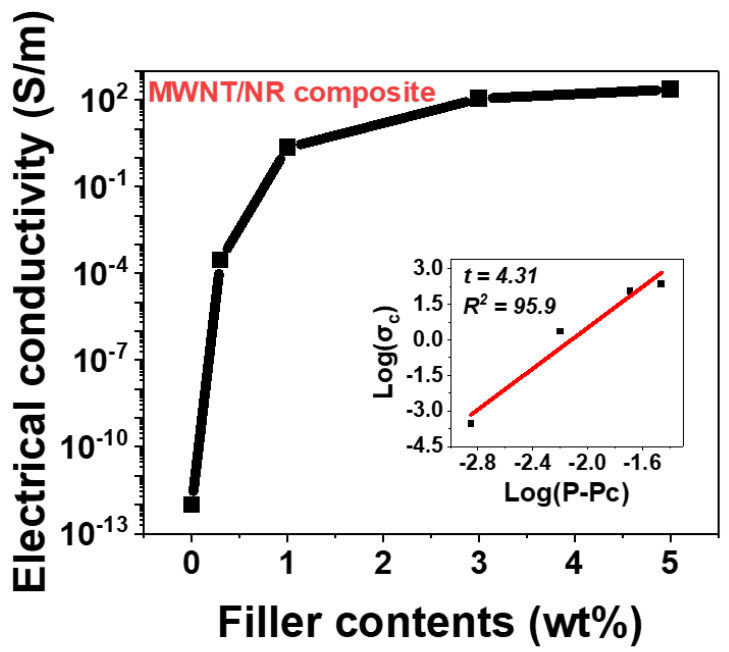
Electrical conductivity of MWNT/NR composites as function of contents (wt.%) and electrical percolation threshold of MWNT/NR composite. Inset image is a loglog plot: relationship between electrical conductivity of the composites and (P−Pc).

The electrical conductivity of the composite mainly depends on the content of the conductive filler, the aspect ratio, and dimensions. Because the electrical pathway is formed by the contact points between conductive fillers and the tunneling effect [32,33], 1D fillers are more effective in constructing an electrical network than 0D and 2D fillers [2]. Therefore, 1D fillers can allow the insulating polymer to be conductive, even with small contents, which 0D and 2D fillers cannot. This is referred to as the electrical percolation phenomenon [34,35]. In this study, we calculated the electrical conductivity and electrical percolation threshold of MWNTs in the NR matrix. The electrical conductivity of the composite was calculated using the following equation:

(1) $$\sigma_{conductivity}=\frac{l}{RA}$$

where $\sigma_{conductivity}$ ($\sigma_{c})$ is the electrical conductivity of the composite, l is the distance between the metal electrodes, R is the resistance of the composite, and A is the cross-sectional area of the sample. Based on Equation (1), we measured the electrical conductivity of the MWNT/NR composite at each concentration. As the number of MWNTs increases, the contact points between MWNTs increase, which leads to higher electrical conductivity, as shown in Figure 3. Owing to percolation, the MWNT/NR composite became electrically conductive at certain points. This is referred to as the electrical percolation threshold, measured at 0.1 wt.% in this study. According to the electrical percolation equation, the relationship between filler content and electrical conductivity can be determined as follows:

(2) $$\sigma_{c}=\sigma_{0}{\left(P-P_{c}\right)}^{t}\;where\;P>P_{c}$$

where $\sigma_{0}$ is a constant, P is the filler content, $P_{c}$ is the percolation threshold, and t is the critical index. Using Equation (2), we calculated *t* to be 2.51, similar to previous studies [34]. However, when the MWNT content exceeds 3 wt.%, the electrical conductivity gradient decreased. This implies that 3 wt.% is sufficient to form an electrical network.

Therefore, even if more MWNTs are present in the NR matrix, the electrical conductivity curve saturates at a certain value.

### 3.3. Mechanical Properties

**Figure 4:**
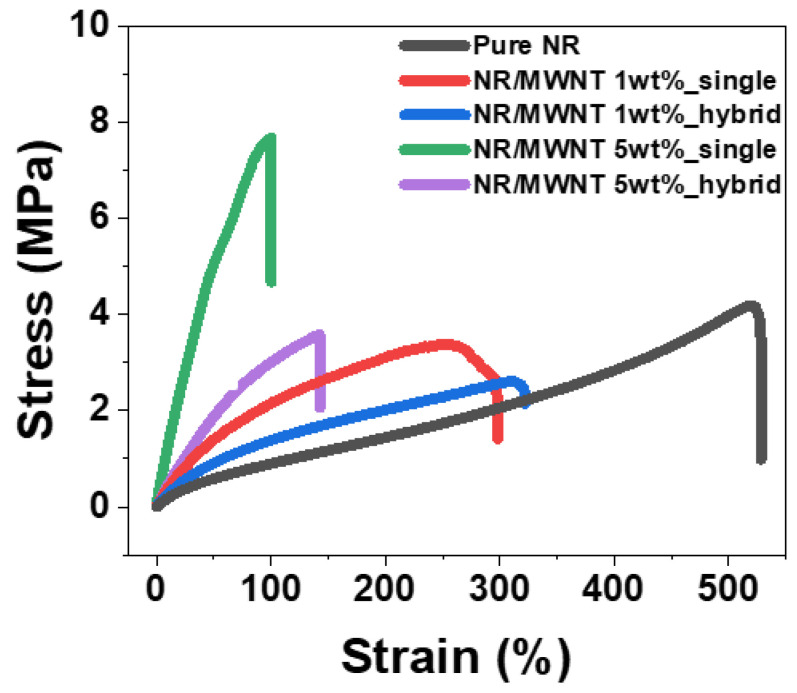
Strain–stress curve of pure NR and MWNT/NR composite according to each concentration and structural formation pathway.

Tensile tests were conducted to analyze the mechanical properties according to the MWNT content and structure form, and the results are presented in Figure 4. There are two types of interaction between nano-fillers and a matrix polymer: covalent and non-covalent interactions [36,37]. Since these different interactions could affect the composite’s mechanical properties, considering the interaction between the filler and matrix is essential. In our case, there are no active functional groups between the MWNT and the NR. Therefore, only physical interactions were considered when discussing the composite’s mechanical properties. When CNT is combined with a polymer, the tensile stress is transferred to the interface between the CNT and the polymer and converted to shear stress. Therefore, CNT composites are more mechanically strengthened than pure polymers [38]. Consequently, as the MWNT content increased, Young’s modulus of the composites increased, but the elongation decreased, owing to the trade-off relationship between the modulus and elongation [39]. In addition, we analyzed the effect on the mechanical properties of sealing the pure NR layers in a single composite. Because pure NR has a lower modulus and better elongation than a single composite, the hybrid composite not only has a moderate modulus, but also increased elongation [3]. In addition, pure NR layers may prevent surface cracks from tensile strain on the conductive composite layer, owing to the excellent adhesion between the interfaces. These results are presented in Table 1. Based on the elongation analyzed through the tensile test, the range for the stretching/releasing dynamic strain sensing test of all samples was set.

### 3.4. Dynamic Strain Sensing Properties

#### 3.4.1. Hysteresis and Plastic Deformation

**Figure 5:**
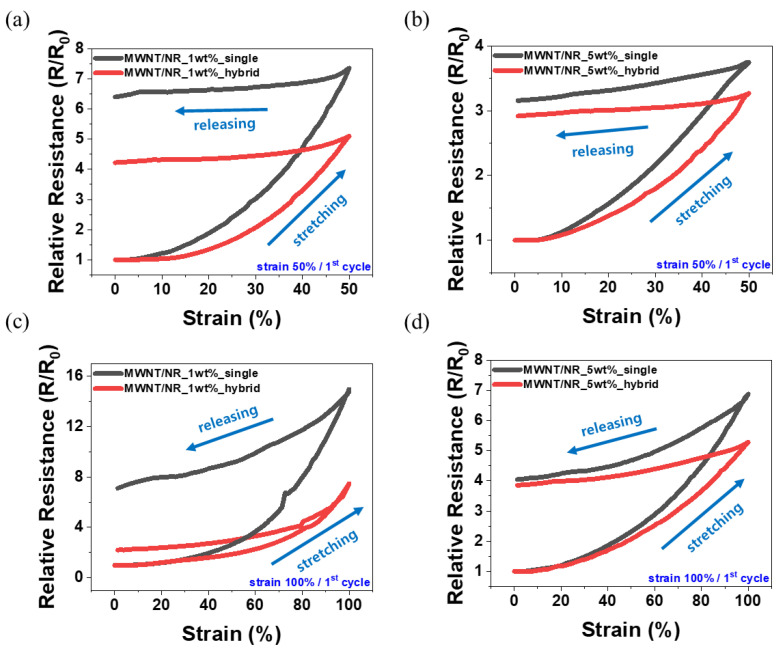
Hysteresis curve of 50% strain range for (**a**) 1 wt.% single composite and (**b**) 5 wt.% hybrid composite. Hysteresis curve of 100% strain range for (**c**) 1 wt.% single composite and (**d**) 5 wt.% hybrid composite.

The effects of CNT content and structure formation on hysteresis during the dynamic strain sensing test were analyzed. All samples were stretched to 50% and 100% tensile strains, and hysteresis analysis was performed in terms of the relative resistance change (R/R_0_) that occurred in the first cycle. According to Figure 5a,b, the hysteresis of 1 wt.% MWNT/NR and 5 wt.% MWNT/NR decreased from 640% to 422%, and 315% to 292, respectively, at 50% strain, when a hybrid structure was adopted. Hysteresis occurs because the electrical network of the CNTs that are initially formed is deformed [17,22]. Therefore, when the strain range is large and the CNT content is small, the deformation of the electrical pathway is large, which leads to a large hysteresis. However, sealing with an NR layer can reduce the hysteresis in a single composite. Further, owing to the excellent adhesion between the NR and composite layers, the hybrid structure can prevent cracks on the composite surface. In addition, the elastic recovery characteristics of pure NR can control the plastic deformation of the conductive layer, which can reduce hysteresis. Plastic deformation is a phenomenon whereby plastic does not return to its initial length when it relaxes after stretching. Because CNTs in elastomers can interfere with the mobility of NR chains when relaxed after strain, plastic deformation of CNT composites is an inevitable issue. In the case of our hybrid structure, it exhibited excellent adhesion between the interfaces, thus reducing plastic deformation. Therefore, hysteresis can be reduced by reducing plastic deformation. The reduced plastic deformation, based on content and structure, is presented in Table 2. Because such hysteresis dominates the CNT content and the initially formed electrical network, the effect of introducing the hybrid structure may not be exhibited well for 5 wt.% MWNT/NR. Similar trends are observed in Figure 5c,d. The hysteresis of 1 wt.% MWNT/NR and 5 wt.% MWNT/NR decreased from 710% to 220%, and 405% to 385%, respectively, at 100% strain in the hybrid structure. That is, the lower the CNT content and the higher the tensile rate, the greater the hysteresis reduction effect of the hybrid structure. However, the higher CNT content was relatively insufficient, owing to the well-constructed initial electrical network.

#### 3.4.2. Wide Sensing Range

**Figure 6:**
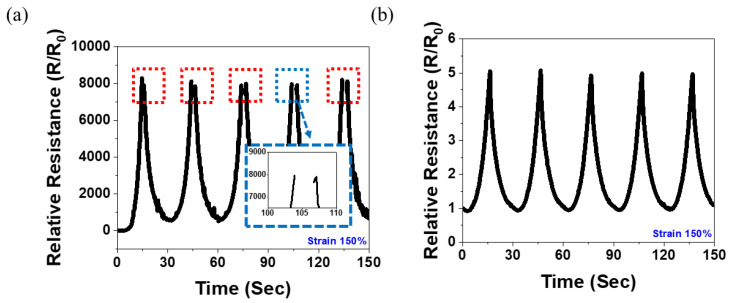
Dynamic strain-sensing curve for (**a**) 1 wt.% single composite strain at 150% and (**b**) 1 wt.% hybrid composite strain at 150% under 5 cycles.

In this study, we analyzed the effect of the hybrid structure on the sensing range of each MWNT content. Figure 6 shows the results for the 1 wt.% MWNT/NR composite. Because the 1 wt.% content is small, the electrical network may be relatively easily destroyed by external strain. Therefore, the tensile strain increases the electrical resistance of the composite, which leads to an increase in the relative resistance. Figure 6a presents the 150% stretching/releasing cycle test of the 1 wt.% MWNT/NR single composite at 30 s/cycle rates. The relative resistance increased to approximately 130%; however, above that, the electrical network was destroyed and an unrecognizable region appeared. This is shown in the inset of Figure 6a. However, the measurement at 150% strain was uniformly performed in the hybrid system (Figure 6b). Although the change in the relative resistance of the hybrid structure decreased, R/R_0_ could be stably controlled, and sensing could be possible in a strain range of 150%, which would not be achieved in a single composite.

**Figure 7:**
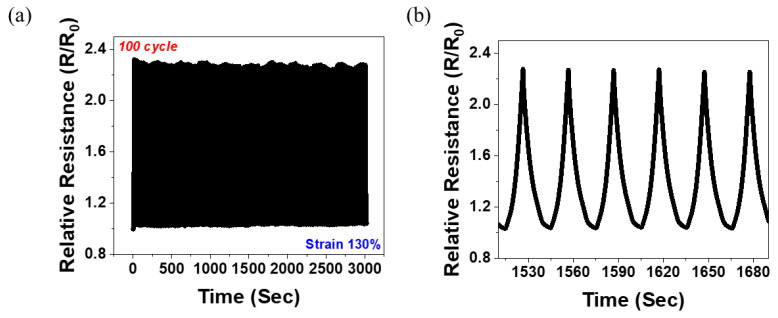
Dynamic strain-sensing curve for (**a**) 5 wt.% hybrid composite strain at 130% under 100 cycles and (**b**) strain-sensing curve from 50th cycle to 56th cycle. (For (**a**,**b**), 20 pre-cycles were conducted before the cyclic test.)

In Figure 7, the dynamic strain-sensing curve of the 5 wt.% MWNT/NR is shown. According to the results presented in Figure 4, the 5 wt.% MWNT/NR single composite broke by ~105%, while the hybrid structure broke by ~142%. That is, because the hybrid structure can be measured in a strain range that was impossible in the single composite, the result of repeating 130% stretching/releasing under 100 cycles was possible, as shown in Figure 7a. The cyclic tests were conducted at a rate of 30 s/cycle. Figure 7b shows that the sample can be operated stably. Furthermore, the 5 wt.% MWNT/NR hybrid structure composite could be a strain sensor with excellent repeatability and durability in the 130% strain range.

#### 3.4.3. Mechanism

**Figure 8:**
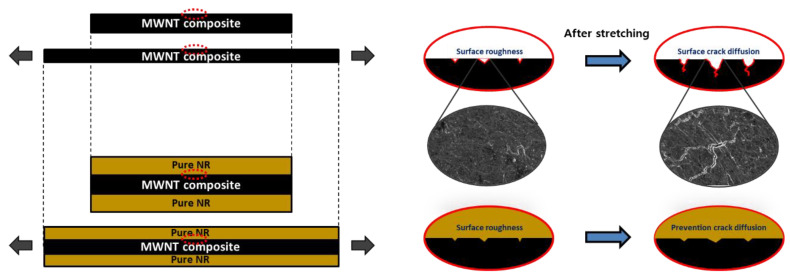
Schematic of the behavior of the surface crack in single- and hybrid-structured MWNT/NR nanocomposite samples under stretching.

The changes in the surface morphology of the single- and hybrid-structured composites during tensile strain are shown in Figure 8. Unlike the ideal model, in the case of a composite manufactured through a heat-pressing process, surface roughness was observed on the surface to a minimal extent. That is, when the single composite was stretched, some defects that had formed on the surface were expanded by tensile stress. This expansion of surface defects can be predicted to lower the strain-sensing range, compared to the results of the ideal model. In addition, the continuous diffusion of surface cracks during repeated strain sensing can further increase the relative resistance (Figure 6). However, in the case of the hybrid composite, the elastomer layer covered the surface of the single composite to fill fine cracks. The elastomer layer can prevent the diffusion of cracks during tensile strength testing, owing to the excellent adhesive property between the interfaces. In addition, because the elastomer layer has a higher elongation than the MWNT composite layer, the adhesive force can be stably maintained even at a high enough strain to break the single layer. The prevention of crack diffusion increases the maximum elongation, and can result in a larger measurable strain range than a single composite. Therefore, the hybrid composite could operate as a wide-range sensor (Figure 7).

## 4. Conclusions

Based on the MWNT content, we analyzed the effect of the hybrid structure, produced by sealing the pure NR layers on the MWNT/NR composite, on the hysteresis and the sensing range of the strain sensor. The adhesion between the pure NR layer and the MWNT/NR composite was excellent, as can be observed from the SEM images. Electrical conductivity, based on the number of MWNTs, was measured using the electrical percolation threshold. The tensile stress test was conducted to set the strain-sensing range, and it was observed that the elongation of the hybrid structure composite increased compared to that of the single composite. Hysteresis analysis was performed at 50% and 100% CNT content, and the hysteresis reduction effect of the hybrid structure was maximized in the case of low CNT content and large tensile range. In addition, the inevitable plastic deformation in a single composite can be reduced in the hybrid structure. Because the pure NR layer holds sufficient surface roughness on the single composite, a strain sensing of approximately 150%, which was not possible in the 1 wt.% MWNT/NR single composite, was possible. Repeatability and durability were excellent in the strain range of 130% in the 5 wt.% hybrid composite. This study confirmed that the pure NR layer can control the structural surface roughness to lower the hysteresis and increase the sensing range. The results of this study show that the characteristics of a strain sensor can be modified through structural deformation. Our conclusions constitute a significant milestone for the future development of strain sensors.

## Figures and Tables

**Table 1:** Mechanical properties of pure NR and MWNT/NR composite.

Mechanical Property	Pure NR	1 wt.%		5 wt.%	
		Single	Hybrid	Single	Hybrid
Young’s modulus [MPa]	1.8	3.7	2.6	13.79	4.6
Elongation at break [%]	528	298	321	105	142

**Table 2:** Degree of plastic deformation of the MWNT/NR composite, based on each concentration and structural formation.

Plastic Deformation	1 wt.%		5 wt.%	
	Single	Hybrid	Single	Hybrid
at strain 50%	8.54	4.01	17.12	10.19
at strain 100%	12.81	9.53	18.62	11.68

## Data Availability

Not applicable.
